# A Dominant Mutation in G_α_s‐Protein Increases Hair Pigmentation

**DOI:** 10.1111/pcmr.70025

**Published:** 2025-05-12

**Authors:** Philip S. Goff, Peter Budd, Darren W. Logan, Margaret Keighren, Marta Cantero, Lisa McKie, Lluis Montoliu, Ian J. Jackson, Elena V. Sviderskaya

**Affiliations:** ^1^ School of Health and Medical Sciences City St George's, University of London London UK; ^2^ MRC Human Genetics Unit Institute of Genetics and Cancer, University of Edinburgh Edinburgh UK; ^3^ Department of Molecular and Cellular Biology National Centre for Biotechnology (CNB‐CSIC) Madrid Spain; ^4^ Centre for Biomedical Network Research on Rare Diseases (CIBERER‐ISCIII) Madrid Spain

**Keywords:** ASP, G‐alpha S, MC1R, McCune Albright syndrome, melanocytes, NDP‐MSH, pigmentation

## Abstract

We have identified a chemically induced mouse mutation which increases the eumelanic hair pigmentation. We identify a coding mutation, A3533G, resulting in an amino acid substitution Y1133C, in the *Gnas* gene encoding the G_α_s subunit of the tripartite G‐protein, consistent with an activation of signalling via MC1R. In addition heterozygous mutant females are significantly lighter than wild type littermates. In cultured melanocytes, derived from mutant mice crossed to C57BL6 mice carrying *Cdkn2a* ^*tm1Rdp*^, basal pigmentation is higher than wild type melanocytes derived from litter mates. However, the addition of exogenous NDP‐MSH does not increase pigmentation in mutant melanocytes in contrast to the pigmentation response of non‐mutant melanocytes. The mutant and wild type cells respond in the same way to agouti signalling protein (ASP), consistent with ASP signalling mediated through a pathway other than G_α_s‐protein.

## Graphical abstract

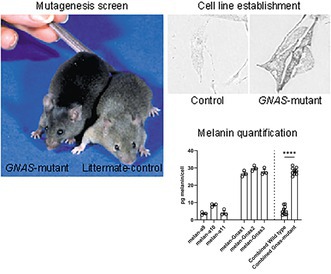

**Summary**

- The mutant G_α_s protein leads to increased pigmentation in melanocytes in culture, which is not further enhanced by stimulation of MC1R, suggesting melanogenesis is saturated in the mutant.

Study of mouse mutations has enabled the dissection of molecular pathways in melanocyte development and cell biology and mutagenesis screens have discovered additional mouse mutations that inform on melanocyte biology. Chemical mutagenesis usually produces point mutations which can result in phenotypes different to a complete loss of function. Screens have identified roles for genes not previously found as pigmentation mutations (Fitch et al. 2003; Tsipouri et al. 2004). Notable are dominant, activating mutations in genes encoding the G‐protein α subunits, GNAQ and GNA1, coupled to EDNRB, resulting in dark skin, (Van Raamsdonk et al. 2004). The mutations enhance the proliferation of melanoblasts prior to their migration to the epidermis and this darkens non‐hairy skin by greatly increasing dermal pigmentation.

In a wide‐ranging mutagenesis screen (Nolan et al. 2000) we have identified a semi‐dominant mutation named *Goth*, which results in an increase in the eumelanic segment of the hair, with a concomitant decrease in phaeomelanin, reminiscent of mutations resulting in enhanced signalling through melanocortin 1 receptor (MC1R) (Figure 1A). The founder mouse was a Balb/c X C3H hybrid which we subsequently backcrossed to C3H.

**FIGURE 1:**
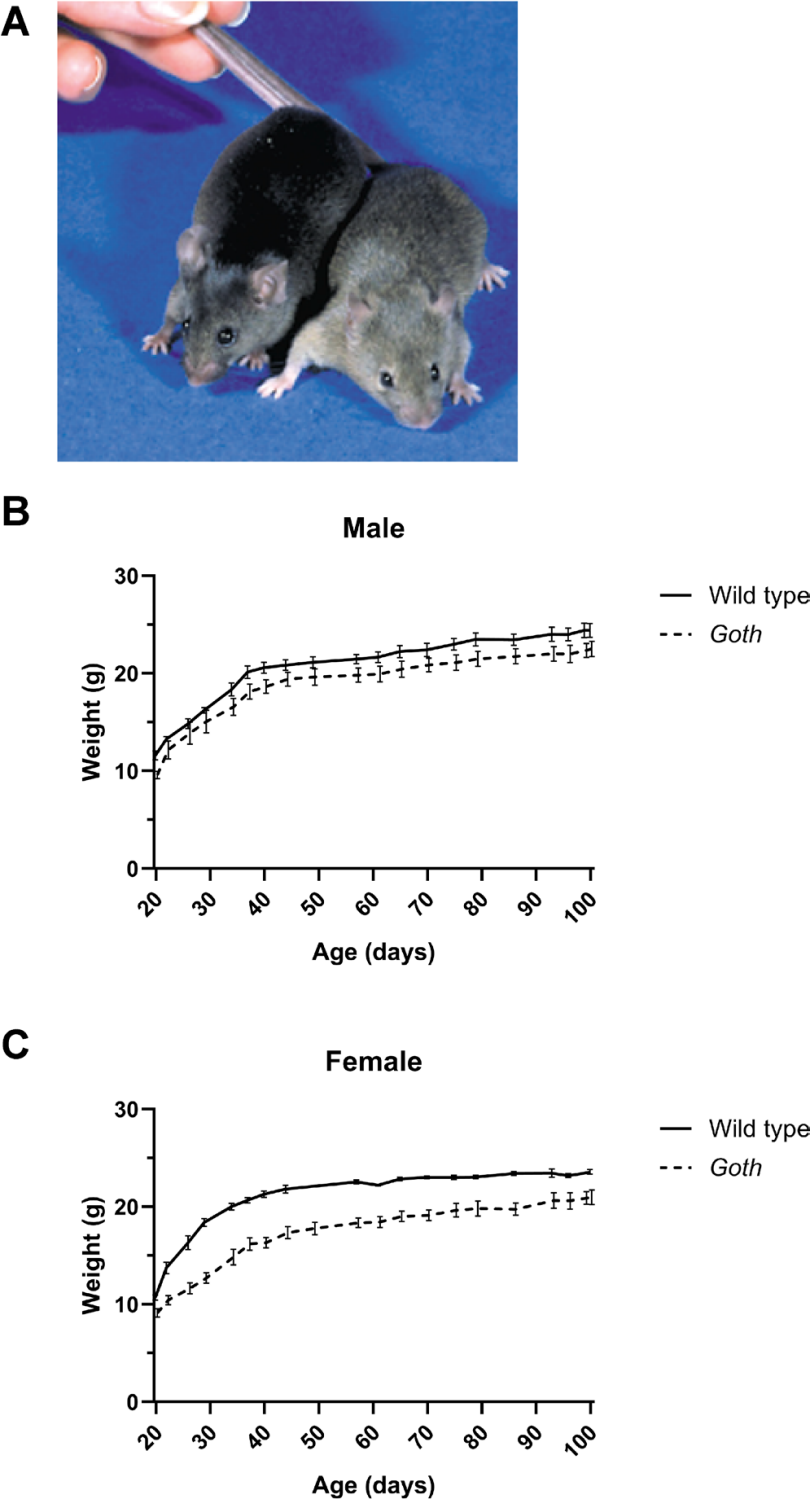
(A) Wild‐type (right) and *Goth* mutant (left) mice. (B) Growth curves of male *Goth* mutant and wild type mice. Weights of 6 wild type and 7 *Goth* measured at intervals between 20 and 100 days of age. Data is expressed as mean weight in grams ± SEM. (C) Growth curves of female *Goth* mutant and wild type mice. Weights of 3 wild type and 7 mutants, expressed as above. Data is in Table S1.

Pigmentation of hair is subject to regulation by a pair of ligands acting through MC1R. Activation by α‐melanocyte stimulating hormone (α‐MSH) promotes the synthesis of dark eumelanin whilst the action of agouti signalling protein (ASP) promotes the synthesis of the paler, yellow or red, phaeomelanin (Furumura et al. 1996). Mouse dorsal hairs typically have a phaeomelanic stripe against a eumelanic background due to a pulse of ASP expression during hair growth (Vrieling et al. 1994). Loss of function mutations in MC1R result in hair that is phaeomelanic throughout (Robbins et al. 1993). In contrast mice with dominant, activating point mutations in MC1R have darker coats (Furumura et al. 1996).

MC1R is a G‐protein coupled receptor. Ligand free or α‐MSH stimulated signalling is thought to go via coupling to the G_α_s, stimulatory G‐protein α subunit, which activates adenylyl cyclase to produce cAMP (Wolf Horrell et al. 2016). Downstream of cAMP includes the activation of protein kinase A (PKA) which leads to phosphorylation of, among others, the Melanogenesis Associated Transcription Factor (MITF) and the melanogenic enzyme, tyrosinase (Bentley et al. 1994; Bertolotto et al. 1998; Price et al. 1998). MC1R also signals independent of G proteins and cAMP via cross talk with the receptor tyrosine kinase, KIT, to activate ERK kinases (Herraiz et al. 2009).

We mapped the *Goth* mutation initially in a backcross to C3H with a genome wide panel of markers (Thaung et al. 2002) followed by refinement using SNPs to ~4 Mb between rs27603062 and rs28261301. There are at least 34 validated genes in this interval. However, given its known role in melanocyte signalling we regarded *Gnas*, mapping in the candidate region and encoding the G_α_s subunit of the tripartite G‐protein, to be a likely candidate. Sequencing identified a single base change A to G at base 3533 of the coding region, changing amino acid 1133, tyrosine to cysteine, four residues from the C‐terminus changing Y‐E‐L‐L to C‐E‐L‐L. The *Goth* phenotype remains associated with the *Gnas* variant after more than 15 crosses and backcrosses to C3H. When crossed onto recessive yellow (*Mc1r* ^*e*^) mice the *Goth* mutation had no effect; the mice were phaeomelanic, indicating that the activated signalling pathway required interaction between the mutant G_α_s protein and MC1R.

Using molecular markers we genotyped intercross litters, and found no homozygous animals after weaning (0 from 22 offspring typed). However homozygotes were identified in intercross litters typed at embryonic day 16.5. We conclude the mutation is lethal at late embryonic or early post‐natal stages (although we cannot exclude the possibility that there is another recessive lethal mutation closely linked to *Goth*). Although it is biallelically expressed in most tissues, the *Gnas* gene shows parent‐of‐origin imprinting in some cell types (Hayward, Kamiya, et al. 1998; Hayward, Moran, et al. 1998). We observed the *Goth* phenotype in offspring with both paternal and maternal transmission.

The human disease, McCune‐Albright Syndrome is caused by somatic, mosaic, activating mutations of *GNAS* and includes dark pigmented patches on the skin, as well as bone and endocrine symptoms (Schwindinger et al. 1992; Weinstein et al. 1991). The mutations are never seen to be transmitted through the germ‐line suggesting embryonic lethality even as heterozygote. Preliminary screening of the *Goth* mice revealed no additional phenotype, however, we asked if the mutation had any impact on growth of the mice. We weighed mice carrying the wild type or mutant *Gnas* from 20 to 100 days with data analysed by a linear mixed model analysis, fit by REML using R Studio (Version 4.4.1) with genotype, sex and time as fixed effects and individual mice as random effects. *t*‐tests used Satterthwaite's method. Raw data table of mice weights are in Table S1. Mutant and wild‐type male mice did not show a statistically significant difference, although there was a trend for mutants to be smaller (*p* = 0.106, Figure 1B). However, mutant female mice consistently weighed significantly less than their litter mate counterparts (*p* = 0.000359, Figure 1C). It is possible that the reduced growth seen in the mutant mice could be due to enhanced signalling through the melanocortin 4 receptor (MC4R), which controls feeding behaviour and weight homeostasis (Huszar et al. 1997; Vaisse et al. 1998; Yeo et al. 1998). Mice and humans with loss of function mutations of MC4R show enhanced weight gain and variants in MC4R are associated with resistance to obesity (Lotta et al. 2019). In fact knockout of *Mc4r* has a greater effect on weight in female mice than in males, mirroring in the opposite direction the sex differences we see (Huszar et al. 1997). However it is also possible that the effects of the mutation are not mediated through MC4R but are due to reduced embryonic or neonatal growth due to defects in other cellular systems.

To explore the pigmentation phenotype in more detail, we crossed the *Goth* mutation into mice homozygous for *Cdkn2a* ^*tm1Rdp*^ (Lavado et al. 2005), a loss of function mutation of *Arf/Ink2a*, and derived melanocyte cell lines from heterozygote mutant and wild‐type littermates, as described in previous studies (Alzahofi et al. 2020; Goff et al. 2023; Sviderskaya et al. 2002). We assayed 3 independent cell lines of each genotype, each line measured in triplicate, which can be obtained from the Functional Genomics Cell Bank at City St George's, University of London. Upon visual inspection by phase‐contrast and bright‐field microscopy, untreated *Gnas*‐mutant melanocytes were hyperpigmented compared to littermate control melanocytes (Figure 2A). The morphology of littermate control melanocytes and *Gnas*‐mutant melanocytes, as visualised by enlarged boxed regions accompanying each low magnification image, does not appear to vary, with bi‐ and tri‐polar cells observed, although differences in pigmentation between the two genotypes are visible in the bright field images. Similarly, treatment with NDP‐MSH of either genotype does not objectively affect the morphology. A quantitative melanin content assay (similar to that used by (Oancea et al. 2009) except melanin is normalised to total cell number) (Figure 2B) showed that melanin content of *Gnas*‐mutant melanocytes is significantly higher (using two‐tailed Student's *t*‐test) than that of littermate controls consistent with a high level of constitutive MC1R‐signalling in *Gnas*‐mutant melanocytes.

**FIGURE 2:**
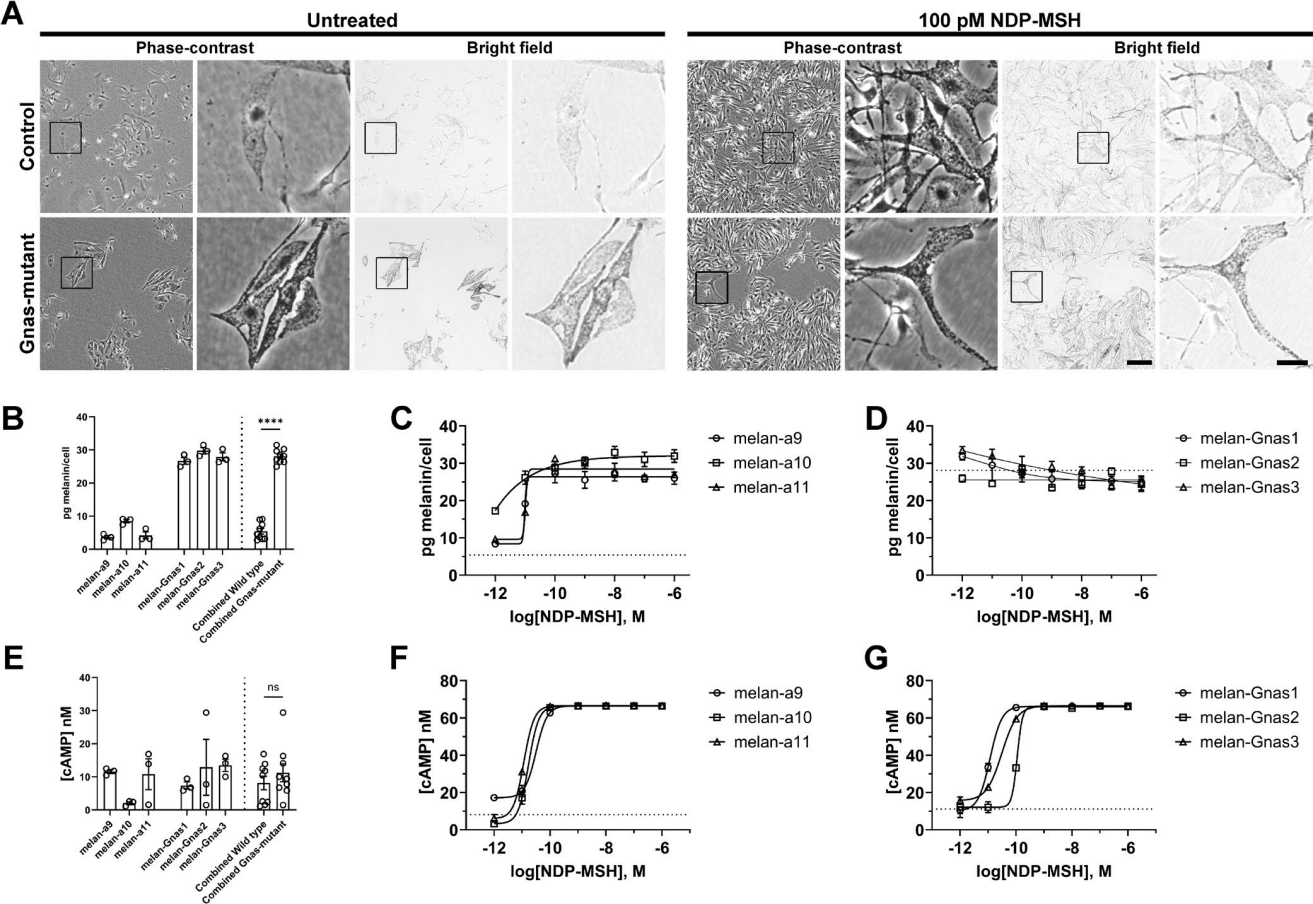
(A) Representative paired phase‐contrast and bright field microscopy images of wild‐type control (melan‐a9) and *Gnas*‐mutant (melan‐Gnas1) melanocytes at steady‐state or treated with 100 pM NDP‐MSH for 7 days. Scale bar = 100 μm. Boxed regions are enlarged 5x next to the corresponding image for both phase‐contrast and bright field images. Scale bar of enlarged images = 25 μm. (B) Comparison of the basal melanin content of wild‐type (melan‐a9, −a10 and ‐a11) and Gnas‐mutant (melan‐Gnas1, ‐Gnas2 and ‐Gnas3). Melanin content was quantified after 7 days in culture, normalised to cell number and expressed as mean pg melanin/cell ± SEM. Data are the mean of 3 technical replicates for each cell line. The average pg melanin/cell were combined for each genotype and compared by two‐tailed t‐test. **** = *p* < 0.0001. Dose–response relationship between melanin content and NDP‐MSH concentration of wild‐type (C) and Gnas‐mutant melanocytes (D). Cells were treated with NDP‐MSH for 7 days, melanin content was quantified, normalised to cell number and expressed as mean pg. melanin/cell ± SEM. Data are the means of 3 technical replicates for each cell line. A four‐parameter dose–response curve is shown for each cell line. Dotted line is the average pg melanin/cell of all three untreated wild type melanocytes lines from (B). (E) Comparison of the basal cAMP concentration of wild‐type (melan‐a9, ‐a10 and ‐a11) and Gnas‐mutant (melan‐Gnas1, ‐Gnas2 and ‐Gnas3) melanocytes. Absolute cAMP concentration quantified by luminescence‐based assay and expressed as nM cAMP ± SEM. Data are the mean of 3 technical replicates for each cell line. Mean basal cAMP concentration of the three wild‐type lines (8.16 ± 2.36 nM) were not significantly different to the three *Gnas*‐mutant lines (11.26 ± 2.29 nM), (*p* = 0.42 by unpaired two‐tailed *t*‐test). The dose‐dependent effects of NDP‐MSH on intracellular cAMP concentration of wild‐type (F) and Gnas‐mutant (G) melanocytes. Cells were treated with NDP‐MSH for 20 min, with absolute cAMP concentration quantified by luminescence‐based assay and expressed as nM cAMP ± SEM. Data are the means of 3 technical replicates for each cell line. A four‐parameter dose–response curve is shown for each cell line. The average basal cAMP concentration for each genotype is represented by the dotted line.

We addressed the effects of MC1R signalling on wild‐type and *Gnas*‐mutant melanocytes using 4‐Norleucine, 7‐D‐phenylalanine‐α‐melanocyte‐stimulating hormone (NDP‐MSH), a potent synthetic analogue of α‐MSH (Sawyer et al. 1980). Wild type cells showed a dose–response relationship between NDP‐MSH and melanin content of wild‐type melanocytes (Figure 2C) with a maximal effect observed using 100 pM NDP‐MSH (see right panel of Figure 2A for effect of 100 pM NDP‐MSH on visible pigmentation and cell morphology). Concentrations of NDP‐MSH over 100 pM had no further effect on the melanin content suggesting saturation of MC1R signalling.

In contrast, NDP‐MSH did not increase the melanin content of *Gnas*‐mutant melanocytes compared to the basal values (Figure 2A,D). The high basal levels of melanin in *Gnas*‐mutant melanocytes without ligand stimulation through MC1R suggesting that the *Gnas* mutation is affecting the MC1R signalling pathway through increased of ligand‐independent signalling. We suggest that there may be a mechanism to limit the amount of melanin produced within the melanocytes, and the mutant cells reach this limit without stimulation.

The G_α_s subunit encoded by the *Gnas* gene activates adenylate cyclase which increases cAMP production to enhance melanogenesis. Mutations in both murine and human *GNAS* can cause excessive cAMP signalling in osteogenic cells isolated from mutant mice and in areas of fibrous dysplasia of human bone respectively (Saggio et al. 2014; Weidner et al. 2020). Therefore we tested whether the *Gnas*‐mutation increased intracellular cAMP. We assayed absolute cAMP concentrations (determined using the cAMP‐Glo Assay kit (Promega) as per the manufacturer's instructions) in unstimulated (Figure 2E) and NDP‐MSH‐stimulated wild‐type (Figure 2F) and *Gnas*‐mutant melanocytes (Figure 2G). We find cAMP concentration increased in both wild‐type and *Gnas*‐mutant melanocytes in response to NDP‐MSH in a dose‐dependent manner, with a maximal response seen at 100 pM NDP‐MSH for both genotypes. However, the mean basal cAMP concentrations of the three wild‐type lines were not significantly different to the three *Gnas*‐mutant lines (Figure 2E). This lack of increased basal cAMP in the more pigmented mutant cells may involve cAMP compartmentalisation within cells. Under basal conditions, most cAMP is bound to specific sites, likely to avoid unwanted activation of cAMP targets (Bock et al. 2020). These cAMP targets, such as PKA, are “protected” by the activity of localised phosphodiesterases (PDEs) that degrade cAMP. It has also been demonstrated that G‐protein coupled receptors (GPCRs) can signal via localised nanodomains of cAMP, to achieve specificity of the GPCR signalling (Anton et al. 2022) and these localised cAMP nanodomains are able to merge and result in a generalised cellular response at high levels of GPCR signalling. It may be that under basal conditions the constitutive activity of MC1R causes localised regions of high cAMP sufficient to increase downstream signalling but this local elevation may not be detectable using our total cell assay. Upon treatment of *Gnas*‐mutant melanocytes with NDP‐MSH, the local nanodomains of cAMP may merge and lead to a more global cAMP response as previously suggested (Anton et al. 2022).

An alternative reason for the lack of effect of the mutation on basal cAMP is that the mutant G_α_s is signalling through an alternative pathway. The MC1R antagonist ASP acts through an alternative pathway (Hida et al. 2009). Previous work has shown that there is a high level of ligand‐independent MC1R signalling in vivo that can be modulated by ASP (Jackson et al. 2007). We asked whether ASP played a role in the hyperpigmentation phenotype of Goth mice and *Gnas*‐mutant cell cultures through reduced sensitivity of MC1R to antagonism by ASP.

The effect of ASP on the visible pigmentation and morphology of wild‐type and *Gnas*‐mutant melanocytes was inspected by microscopy (Figure 3A). Previous work has shown 10 nM ASP is sufficient to induce phaeomelanogenesis in wild‐type melanocyte cultures (Hida et al. 2009). This was confirmed by dose–response experiments in the present study (Figure S1). Paired phase‐contrast and bright field images show that treatment of both wild‐type and mutant melanocytes with ASP resulted in a more melanoblast‐like phenotype with reduced pigmentation compared to untreated controls, although several pigmented mutant melanocytes remain. The reduction in melanin content in response to ASP was confirmed by quantitative melanin assay in wild type (Figure 3B) and *Gnas*‐mutant (Figure 3C) melanocytes. The effect of ASP on absolute cAMP concentration of wild‐type (Figure 3D) and *Gnas*‐mutant melanocytes (Figure 3E) was quantified. ASP did not affect absolute cAMP concentration compared to untreated controls in either genotype. This is in agreement with other studies that suggest the effect of ASP on pigmentation is independent of cAMP‐signalling (Hida et al. 2009). These data show that the novel *Gnas*‐mutation does not result in a resistance to ASP.

**FIGURE 3:**
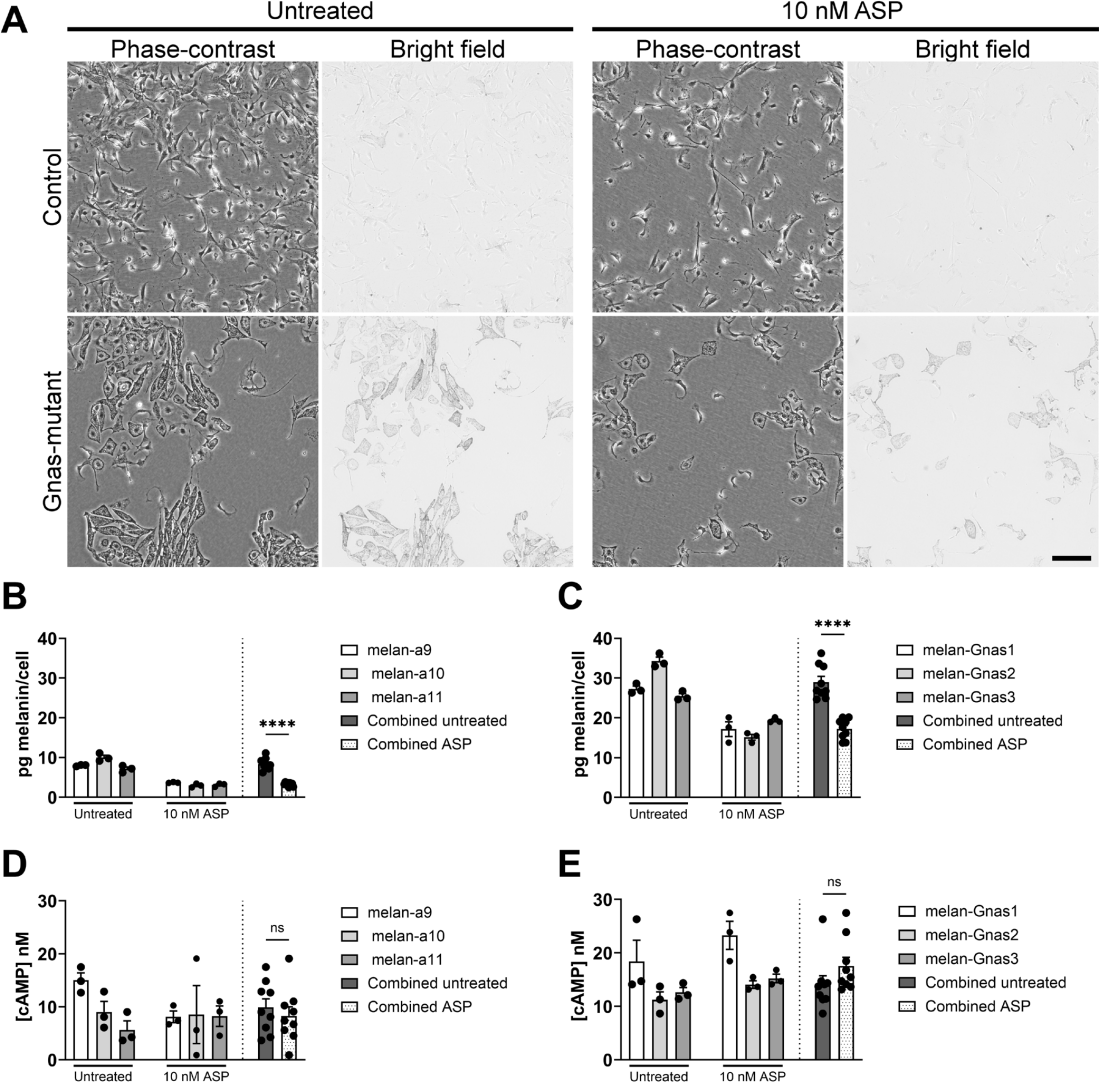
(A) Representative paired phase‐contrast and bright field microscopy images of wild‐type control (melan‐a9) and *Gnas*‐mutant (melan‐Gnas1) melanocytes at steady‐state or treated with 10 nM ASP. Scale bar = 100 μm. The effects of 10 nM ASP on the melanin content of wild‐type (B) and *Gnas*‐mutant (C) melanocytes compared to untreated controls. Melanin content was quantified after 7 days in culture, normalised to cell number and expressed as mean pg melanin/cell ± SEM. Data are the mean of 3 technical replicates for each cell line. The average pg melanin/cell were combined for each genotype and compared by two‐tailed t‐test. **** = *p* < 0.0001. The effects of ASP on intracellular cAMP concentration of wild‐type (D) and *Gnas*‐mutant (E) melanocytes. Cells were treated with 10 nM ASP for 20 min, with absolute cAMP concentration quantified by luminescence‐based assay and expressed as nM cAMP ± SEM. Data are the means of 3 technical replicates for each cell line. A four‐parameter dose–response curve is shown for each cell line. The average basal cAMP concentration for each genotype were combined and compared by two‐tailed *t*‐test. ns, not significant.

The C‐terminus of G_α_s is particularly critical for its function. It is noteworthy that in the mutant replacing the tyrosine four residues from the C‐terminus with cysteine introduces a potential prenylation site (specifically geranygeranylation) which could affect membrane association. The gamma subunit of the trimeric G‐protein is geranylgeranylated as are small G‐proteins such as RAS (Marshall 1993) and this modification is essential for their function. However, the C‐termini of G‐α subunits are shown to be critical in the interaction between these proteins and GPCRs. The specificity of G‐α proteins for the receptor is mediated via the C‐terminal residues. Switching the terminal 5 amino acids between G_α_q and G_α_s permits a normally Gs coupled receptor to stimulate phospholipase C activity and a normally Gq coupled receptor to simulated adenylyl cyclase (Conklin and Bourne 1993).

## Disclosure

Significance statement: This work identifies a novel dominant mutation in G_α_s protein which effects pigment synthesis but other than a reduced growth phenotype, does not have severe detectable effects elsewhere.

## Conflicts of Interest

The authors declare no conflicts of interest.

## Supporting information


**Figure S1.** Dose–response relationship between ASP concentration and melanin content of wild‐type (A) and Gnas‐mutant (B) melanocytes. Cells were treated for 7 days with various concentrations of ASP. Melanin content was quantified, normalised to cell number and expressed as mean pg melanin/cell ± SEM. Data are the mean of 3 technical replicates for each cell line. A four‐parameter dose–response curve is shown for each cell line. The average pg melanin/cell were combined for each genotype and are represented by the dotted line.

**Table S1.** Table containing weights of wild type and *Goth* mice at intervals between 20 and 100 days of age.

## Data Availability

The datasets generated during and/or analysed during the current study are available from the corresponding authors on reasonable request.
