# Supplementary figures and images for: A Dominant Mutation in Gαs‐Protein Increases Hair Pigmentation
Source: Pigment Cell Melanoma Res. 2025 May 12;38(3):e70025. doi: 10.1111/pcmr.70025 (PMC12069967; doi:10.1111/pcmr.70025)

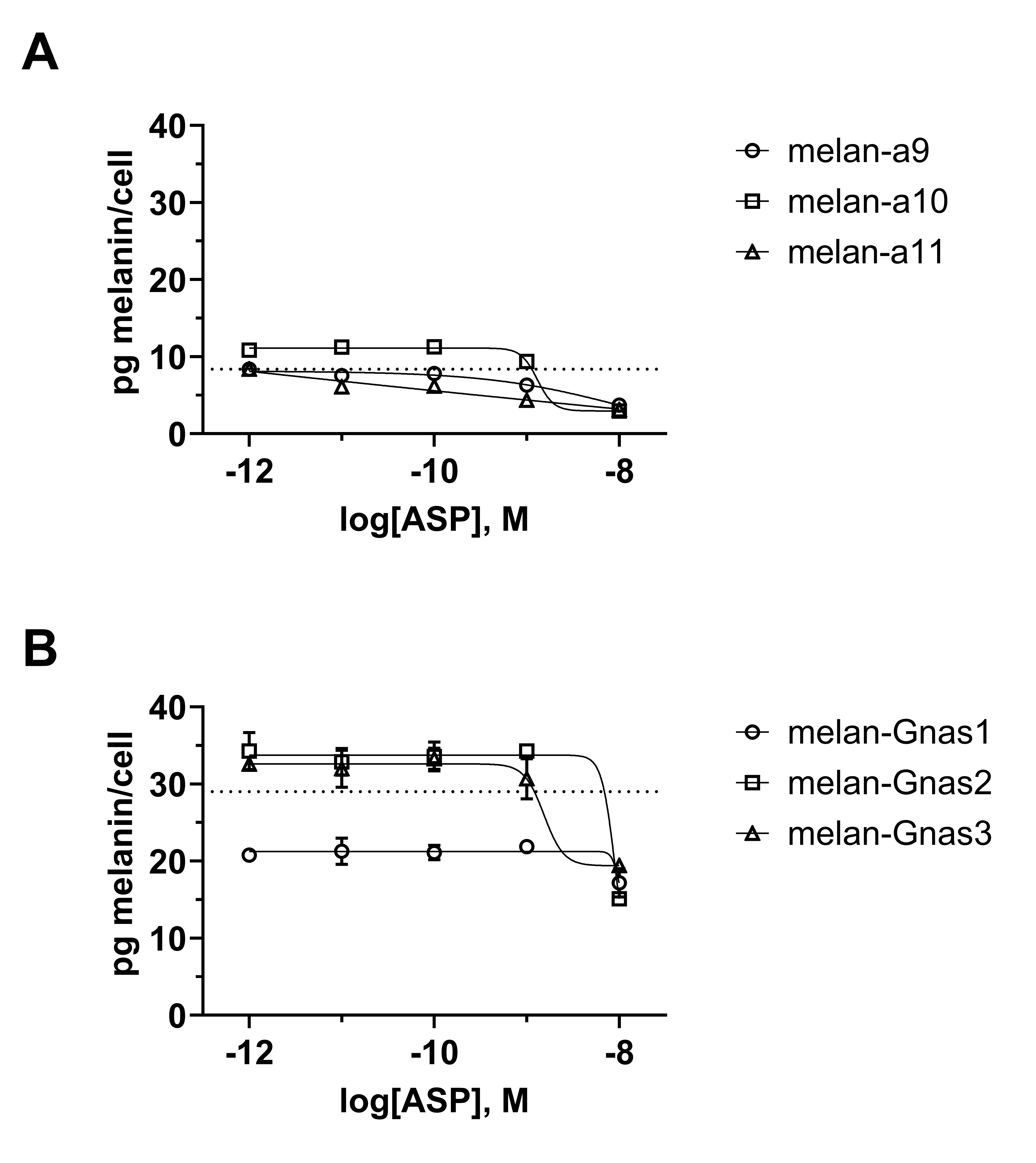

Supplement: Supplementary file 1 — Figure S1. Dose–response relationship between ASP concentration and melanin content of wild‐type (A) and Gnas‐mutant (B) melanocytes. Cells were treated for 7 days with various concentrations of ASP. Melanin content was quantified, normalised to cell number and expressed as mean pg melanin/cell ± SEM. Data are the mean of 3 technical replicates for each cell line. A four‐parameter dose–response curve is shown for each cell line. The average pg melanin/cell were combined for each genotype and are represented by the dotted line. [file PCMR-38-0-s001.tif]
